# Sensitization Prevalence, Antibody Cross-Reactivity and Immunogenic Peptide Profile of Api g 2, the Non-Specific Lipid Transfer Protein 1 of Celery

**DOI:** 10.1371/journal.pone.0024150

**Published:** 2011-08-29

**Authors:** Gabriele Gadermaier, Michael Hauser, Matthias Egger, Rosetta Ferrara, Peter Briza, Keity Souza Santos, Danila Zennaro, Tamara Girbl, Laurian Zuidmeer-Jongejan, Adriano Mari, Fatima Ferreira

**Affiliations:** 1 Christian Doppler Laboratory for Allergy Diagnosis and Therapy, University of Salzburg, Salzburg, Austria; 2 Center for Molecular Allergology, IDI-IRCCS, Rome, Italy; 3 Discipline of Allergy and Immunology, School of Medicine, University of Sao Paulo, Sao Paulo, Brazil; 4 Laboratory of Allergy, Department of Experimental Immunology, Academic Medical Center, Amsterdam, The Netherlands; Centre de Recherche Public de la Santé (CRP-Santé), Luxembourg

## Abstract

**Background:**

Celery (*Apium graveolens*) represents a relevant allergen source that can elicit severe reactions in the adult population. To investigate the sensitization prevalence and cross-reactivity of Api g 2 from celery stalks in a Mediterranean population and in a mouse model.

**Methodology:**

786 non-randomized subjects from Italy were screened for IgE reactivity to rApi g 2, rArt v 3 (mugwort pollen LTP) and nPru p 3 (peach LTP) using an allergen microarray. Clinical data of 32 selected patients with reactivity to LTP under investigation were evaluated. Specific IgE titers and cross-inhibitions were performed in ELISA and allergen microarray. Balb/c mice were immunized with purified LTPs; IgG titers were determined in ELISA and mediator release was examined using RBL-2H3 cells. Simulated endolysosomal digestion was performed using microsomes obtained from human DCs.

**Results:**

IgE testing showed a sensitization prevalence of 25.6% to Api g 2, 18.6% to Art v 3, and 28.6% to Pru p 3 and frequent co-sensitization and correlating IgE-reactivity was observed. 10/32 patients suffering from LTP-related allergy reported symptoms upon consumption of celery stalks which mainly presented as OAS. Considerable IgE cross-reactivity was observed between Api g 2, Art v 3, and Pru p 3 with varying inhibition degrees of individual patients' sera. Simulating LTP mono-sensitization in a mouse model showed development of more congruent antibody specificities between Api g 2 and Art v 3. Notably, biologically relevant murine IgE cross-reactivity was restricted to the latter and diverse from Pru p 3 epitopes. Endolysosomal processing of LTP showed generation of similar clusters, which presumably represent T-cell peptides.

**Conclusions:**

Api g 2 represents a relevant celery stalk allergen in the LTP-sensitized population. The molecule displays common B cell epitopes and endolysosomal peptides that encompass T cell epitopes with pollen and plant-food derived LTP.

## Introduction

In the Mediterranean population, non-specific lipid transfer proteins (LTP) represent important allergens in fruits and vegetables and account for the majority of type I food allergies in adults [1], [2], [3]. Due to their pronounced resistance to thermal and proteolytic treatment [4], they are considered true food allergens and were proposed to mediate sensitization via the gastrointestinal tract [3]. Pru p 3, the major allergen from peach seems to play an important role in LTP-mediated allergy with a sensitization prevalence of 13% and 9.8% in a Spanish and Italian population, respectively [5], [6]. The clinical picture of an LTP-mediated food allergy ranges from local oral allergy syndrome to severe systemic symptoms [7]. We recently identified and characterized an nsLTP1 in celery stalks which was included as Api g 2.0101 in the official I.U.I.S. allergen nomenclature [8]. The natural protein consists of a single isoallergen with a mass of 9024.5 Da. A recombinant protein was produced as non-fusion protein in *E. coli* which presented equivalent physio-chemical and immunological properties as the natural counterpart. In line with other members of the LTP family, Api g 2 shows extreme resistance to gastrointestinal proteolysis and thermal treatment [8]. Notably, the allergen was able to fully refold after heating in acidic environment, a property that was previously also demonstrated for Pru p 3 [8], [9]. In addition to plant-derived foods, LTP have been shown to constitute important allergens in pollen and ever since there is a longstanding debate about the sensitizing source [7]. Generally, the classification of LTP in the field of food allergy turns out to be extremely difficult. On the one hand, LTP fulfill all the requirements of true food allergens, *i.e.* the capability to sensitize via the gastrointestinal tract. On the other hand, they might be regarded as pollen-associated food allergens, as allergenic LTP are also found in plant pollen and associations between pollen and food allergies involving LTP sensitization have been reported [3], [7], [10]. In mugwort and peach allergy, both Art v 3 and Pru p 3 have been proposed as primary sensitizer [11], [12], however at present no evidence suggests a clinical association between sensitization to plant food and pollen LTP.

*Apium graveolens*, celery, is considered one of the most important plant food allergen sources in the adult Central European population [13], [14] and therefore, declaration of products that contain celery is mandatory (European Directive 2007/68/EC, amending Directive 2000/13/EC). Apart from the recently identified nsLTP1, three allergens from *A. graveolens* have been described and characterized at the molecular level, the PR-10 protein Api g 1 [15], the profilin Api g 4 [16], and Api g 5, a member of the flavoprotein family [17]. Among Central European patients, a predominant sensitization to Api g 1 (59%) and Api g 4 (23≥41%) is observed [16], [18]. Although celery stalks are worldwide consumed, there is only limited information on allergens and clinical relevance of the aerial celery tissue, since the majority of studies focused on patients from Central Europe [13], [14], [19], [20], who predominately consume celery tuber (celeriac). Noteworthy, the recently identified LTP1 Api g 2 can be considered a celery stalk-specific allergen, since expression is restricted to the green stalks and was not detectable in the tuber tissue [8].

Clinical allergy to celeriac is frequently associated with sensitization to *Artemisia vulgaris* and *Betula verrucosa* pollen in Central European countries and thus, the terms celery-mugwort and celery-birch syndrome have been established [10]. Association between birch pollinosis and celery hypersensitivity is mainly attributed to Api g 1, a Bet v 1-homologous PR-10 protein [15], [21]. Moreover, Api g 4 and Api g 5 have been mentioned to play a role as cross-reactive molecules in this population [16], [22], [23]. By contrast, a heat stable molecule involved in the celery-mugwort syndrome which might be able to trigger severe allergic reactions in Central European celeriac sensitized patients is not yet conspicuously determined [24].

In this study, we investigated the sensitization prevalence of Api g 2 in allergic patients from the Mediterranean area by testing on a microarray system in parallel with other LTP. The sensitization pattern and IgE cross-reactivity of Api g 2 was investigated in a selected patients' cohort sensitized to Art v 3 and Pru p 3, model allergens for pollen and plant food LTP, respectively. In order to elucidate which molecule has the potential to act as primary sensitizer, we immunized mice with pollen and plant-food derived LTP and determined the patterns of antibody cross-reactivity. Immunogenicity and putative T cell epitopes were determined *in vitro* by simulated endolysosomal degradation assays.

## Results

### IgE profiling of celery stalks, mugwort pollen, and peach LTP in Mediterranean patients

**Figure 1:**
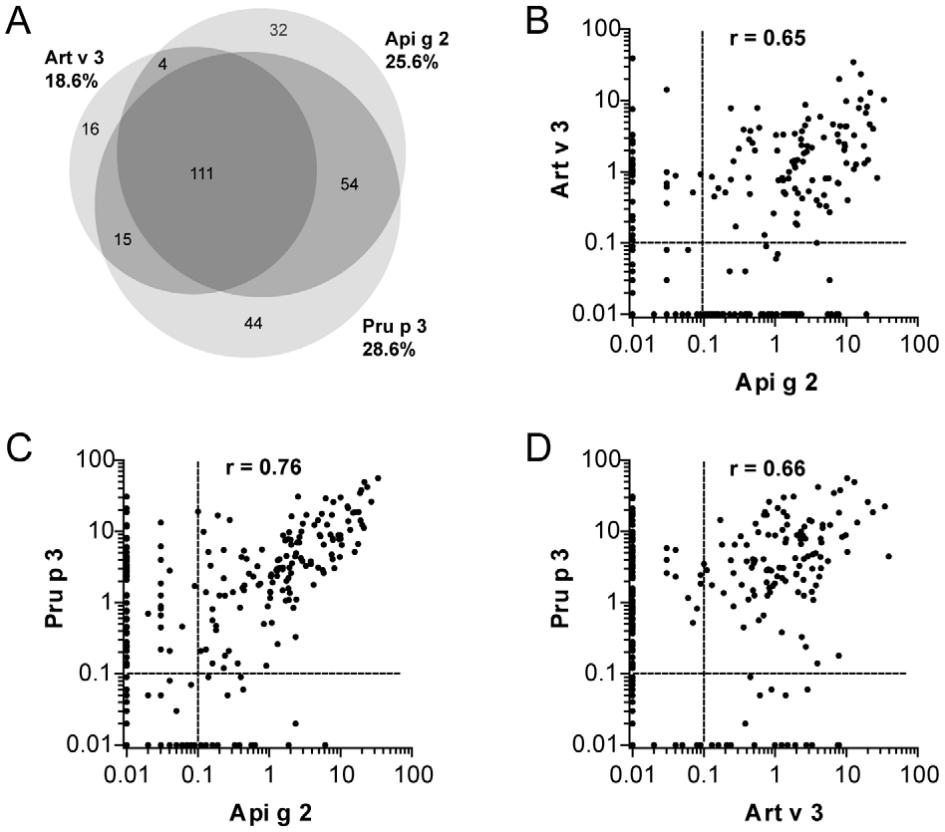
Sensitization to celery, mugwort pollen and peach LTP in a Mediterranean population. 786 individuals were screened for IgE reactivity to rApi g 2, rArt v 3, and nPru p 3 using an allergen microarray and co-sensitization patterns are shown in the Venn diagram (A). Statistical significant correlations of allergen specific IgE reactivity presented as kUA/L (p<0.0001) were observed for Api g 2, Art v 3 and Pru p 3 using the Spearman correlation test (B-D).

To assess the sensitization prevalence of the novel celery stalk allergen in an allergic Mediterranean population, sera of 786 subjects were tested *in vitro* by an experimental ISAC microarray. Among them, 25.6% displayed specific IgE against celery stalk LTP, 18.6% were sensitized to mugwort pollen LTP, and 28.6% to peach LTP. The majority of patients (n = 111) reacted to all 3 LTPs, and associated reactivity of Api g 2 to Pru p 3 and Art v 3 was observed in 54 and 4 individuals, respectively (Figure 1A). Isolated IgE reactivity to Api g 2 was observed in 32 patients, while 16 and 44 patients exclusively reacted to Art v 3 and Pru p 3, respectively. In sensitized individuals, the average IgE reactivity to Api g 2, Art v 3, and Pru p 3 was 1.03 kU_A_/l, 1.12 kU_A_/l, and 2.59 kU_A_/l, respectively. As shown in Figure 1B-D, significant correlations (p<0.0001) in IgE-reactivity were observed between all three LTP under investigation.

### Symptoms and IgE reactivity of patients sensitized to plant food and pollen LTP

Defining Art v 3 and Pru p 3 as model allergens for pollen and food LTP sensitization, we selected a cohort of 32 Api g 2-positive subjects that additionally displayed *in vitro* IgE-reactivity to mugwort pollen and peach LTP. Demographic data, clinical manifestations to the respective sources and IgE reactivity to Api g 2, Art v 3, and Pru p 3 are given in Table 1. In this selected cohort, ten patients (P1-10) reported allergic reactions upon consumption of celery stalks which predominately presented as oral allergy syndrome (80%). In addition, angioedema was observed in 2 patients and anaphylaxis in 1 patient. However, twelve individuals (P11-22) could not unambiguously report about clinical manifestations or tolerance upon consumption of raw celery stalks since they were not exposed, routinely cooked the vegetable as a precaution or actively avoided consumption due to previously severe allergic reactions to other LTP containing food sources. To investigate a possible relevance of other *A. graveolens* allergens in our patients' cohort, sensitization to Api g 1 was derived from a microarray testing revealing a positive IgE reactivity for patient P1 and P29. To give further information on potentially important cross-reactive molecules, profilin sensitization of two patients (P1 and P31) is denoted in Table 1. Notably, among the ten patients with clinical symptoms to celery stalks, only one patient (P1) showed additional sensitization to *A. graveolens* allergens. Thus, for the remaining subjects, Api g 2 might be involved in a clinical manifestations of celery stalk allergy, even though sensitization to yet unknown celery allergens cannot be ruled out and would need further investigation. Adverse reactions upon consumption of peach and inhalant allergy symptoms to mugwort pollen were reported by 27 and 18 patients sensitized to Pru p 3 and Art v 3, respectively (Table 1). LTP-specific serum IgE levels were determined in ELISA showing highest *in vitro* reactivity for Pru p 3 which differed significantly from Api g 2 (p<0.001) and Art v 3 (p<0.02) using the Wilcoxon signed rank sum test (Table 1). Interestingly, heat-denaturation for 15 minutes at 95°C at a concentration of ≥1 mg/ml in phosphate buffer resulted in complete abolishment of IgE reactivity to celery stalk and mugwort pollen LTP, whereas residual IgE-binding (range 5.5–62.0%, median 6.9%) to Pru p 3 was still detectable in 50% of patients (data not shown). No unspecific IgE binding to investigated proteins was observed using sera of non-atopic individuals.

**Table 1:** Demographics, *in vitro* sensitization profile and symptoms table.

			Celery stalks		Mugwort pollen		Peach	
Patient	Age	Gender	Api g 2	Symptoms	Art v 3	Symptoms	Pru p 3	Symptoms
P1	10	M	8.6	OAS*,**	12.0	NS	44.4	OAS
P2	43	M	60.8	OAS	13.0	R	56.8	OAS
P3	20	M	15.6	OAS	6.2	R	17.2	OAS.ANG
P4	15	M	7.2	OAS	7.8	R	12.2	OAS
P5	22	M	21.6	ANX, OAS	24.6	NS	59.6	ANX. OAS
P6	35	M	20.0	OAS	17.8	NS	58.2	A, OAS, U
P7	11	F	11.8	OAS	10.0	NS	43.4	OAS
P8	21	M	72.8	OAS	64.2	NS***	73.2	U
P9	23	M	20.6	ANG	67.6	C, R***	54.8	C, R
P10	26	M	9.4	ANG	3.6	C, R	31.2	A, OAS, U
P11	14	M	50.2	Eaten cooked	65.6	NS	59.2	U
P12	15	M	90.8	Eaten cooked	63.8	R	103.4	No exposure
P13	23	M	38.6	Eaten cooked	53.2	NS	90.8	OAS
P14	23	M	42.4	Eaten cooked	31.6	NS	52.6	No exposure
P15	31	F	1.6	Eaten cooked	7.0	NS	28.6	OAS
P16	35	M	137.6	Avoided	77.4	R, A***	212.8	OAS
P17	23	F	11.8	Avoided	11.0	NS	11.2	NS
P18	27	F	4.6	Avoided	4.2	R	44.6	U, ANG
P19	34	M	17.0	Avoided	79.2	R***	14.4	R, AB
P20	54	F	3.6	Avoided	2.2	R	4.2	OAS, ANG
P21	17	F	6.2	No exposure	49.6	NS	78.6	OAS
P22	33	F	3.4	No exposure	2.4	R***	2.0	OAS
P23	56	F	33.8	NS	149.4	R, C, A***	29.0	ANX
P24	39	M	50.4	NS	61.8	NS***	43.8	NS
P25	24	M	35.8	NS	17.8	R	59.6	OAS
P26	14	F	25.6	NS	41.2	NS	63.0	OAS
P27	35	F	9.8	NS	6.6	R	23.4	OAS, AP, A
P28	45	M	39.4	NS	8.8	R, C	80.4	No exposure
P29	25	F	12.8	NS*	62.0	R, C***	26.8	OAS
P30	36	F	66.2	NS	54.2	NS	76.2	AP
P31	54	F	8.6	NS**	2.8	R	51.8	U
P32	43	F	2.0	NS	2.0	R, C	6.0	OAS
	**Mean = 29** **SD = 13**	**F = 14** **M = 18**	**Mean = 29.4** **Median = 18.5**	**S = 10** **ND = 12** **NS = 10**	**Mean = 33.8** **Median = 17.8**	**S = 18** **ND = 0** **NS = 14**	**Mean = 50.4** **Median = 48.2**	**S = 27** **ND = 3** **NS = 2**

Demographics, clinical symptoms and specific IgE (ng/ml serum) to LTP from celery stalks, mugwort pollen and peach determined in ELISA are given for 32 patients.

A, bronchial asthma; ANG, angioedema; ANX, anaphylaxis; AP, abdominal pain; C, conjunctivitis; ND, not definable; NS, no symptoms; OAS, oral allergy syndrome; R, rhinitis; S, symptoms; SD, standard deviation; U, urticaria;

*, Api g 1 sensitization;

**, profilin sensitization;

***, Art v 1 sensitization.

### IgE cross-reactivity in LTP sensitized patients

**Figure 2:**
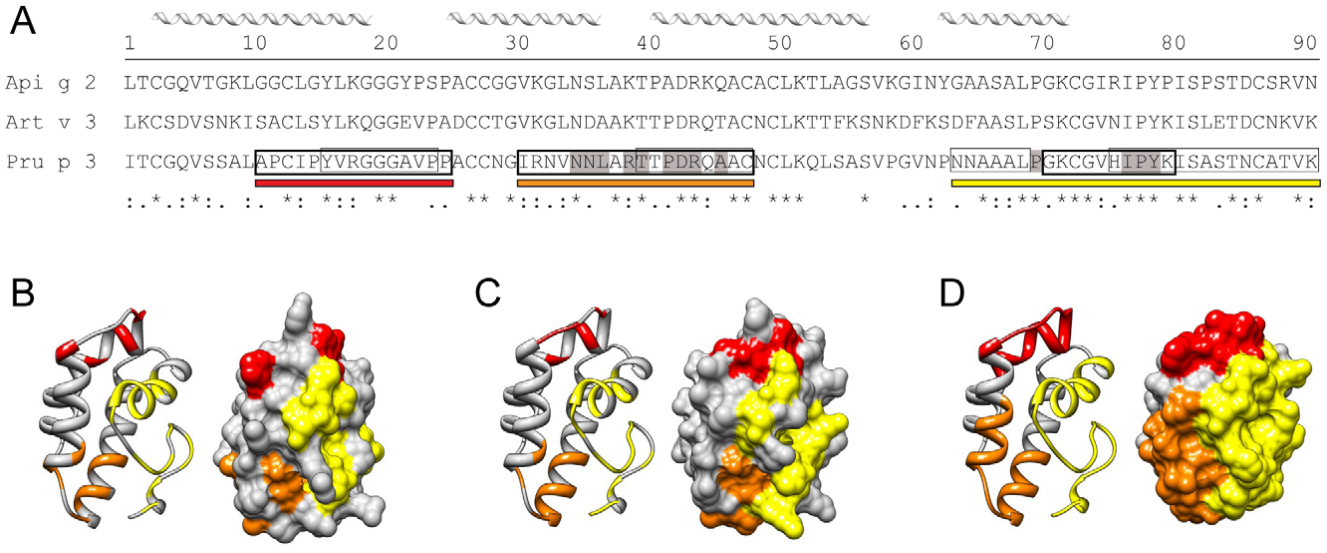
Sequence alignment and homology modeling of LTP. Primary sequence alignment of the mature protein sequences of celery stalk, mugwort pollen and peach LTP (A). Previously identified IgE-binding epitopes of Pru p 3 are labeled with boxes; thick and thin boxed amino acids correspond to peptides identified by Garcia-Casado et al [26] and Borges et al [25], while highlighted amino acids in grey were identified by mimotope mapping [27]. Homology modeling of Api g 2 (B), Art v 3 (C) and Pru p 3 (D) based on the structure of Pru p 3 (pdb: 2B5S). Identified IgE epitopes of Pru p 3 are depicted as red, orange, and yellow bars in the sequence and structure of the molecule. Conserved residues of the Pru p 3 epitopes are depicted colored in the models of Api g 2 and Art v 3.

**Figure 3:**
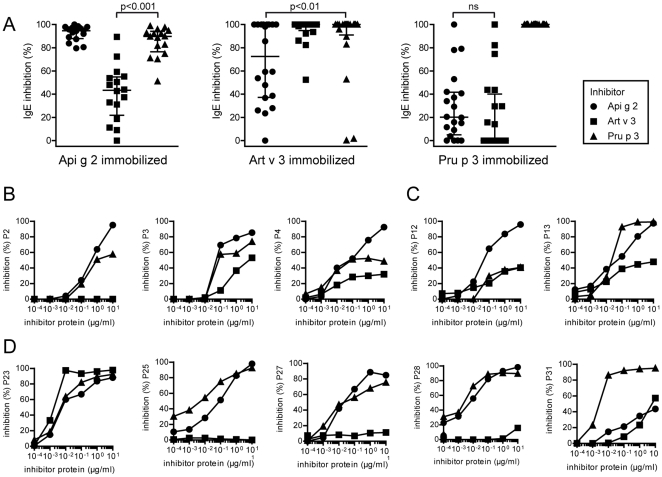
IgE cross-reactivity of LTP sensitized patients. Single point highest inhibition-achievable assays were performed using rApi g 2, rArt v 3, and nPru p 3 for pre-incubation of individual sera (A). Cross-inhibition was tested using the allergen microarray and values are expressed as percentage of IgE inhibition. Data were analyzed using the Wilcoxon signed rank test, the median is shown as solid line and whiskers indicate the interquartile range. Inhibition to solid-phase coated rApi g 2 was evaluated in ELISA using sera of patients with symptoms to raw celery stalks (B) or those consuming solely cooked celery (C), and asymptomatic patients (D). Sera were pre-incubated with increasing concentrations (10^−4^ to 10^1^ µg/ml) of purified LTP and values are given as percentage of inhibition.

The primary sequence identity of Api g 2, Art v 3, and Pru p 3 ranges between 49% and 53%, and amino acid alignments and 3-D models of investigated LTP are depicted in Figure 2. Previously identified linear IgE-binding epitopes of Pru p 3 [25], [26], [27] demonstrate highest sequence identities to Api g 2 (76%) and Art v 3 (82%) within residue 66-82 in the C-terminal epitope (Figure 2). To assess the IgE cross-reactivity of Api g 2, Art v 3, and Pru p 3, inhibition studies were performed using LTP-reactive sera in a single point highest inhibition-achievable assay (Figure 3A). Generally, considerable cross-reactivity was observed among the investigated molecules, however varying degrees of inhibition were achieved using individual patients' sera. Highest cross-inhibition was accomplished with Pru p 3 which was significantly higher than inhibition with Art v 3 and Api g 2 to solid-phase coated Api g 2 and Art v 3, respectively. Generally, IgE reactivity to immobilized Pru p 3 could only be weakly inhibited by Api g 2 (median  =  20.2%) and Art v 3 (median  =  0%). Notably, pre-incubation of sera using Api g 2 for immobilized Art v 3 showed 72.5% (median) and Art v 3 for immobilized Api g 2 presented 43.4% (median) of cross-inhibition, and was thus in average higher than their inhibitory capacity observed towards Pru p 3. Interestingly, a negative correlation (r = −0.842) was found for mugwort and peach LTP inhibition in sera of 3 patients, since Art v 3 could efficiently inhibit Pru p 3 reactivity, but not vice versa. In order to evaluate IgE cross-reactivity to solid phase coated Api g 2 in a dose dependent manner, cross-inhibition experiments were performed using 3 sera of celery stalk allergics (Figure 3B), 2 subjects eating only cooked celery (Figure 3C), and 5 individuals tolerating raw celery (Figure 3D). Notably, patients who showed highest self-inhibition with Api g 2 frequently displayed clinical symptoms upon consumption of celery stalks, while stronger Pru p 3 and/or Art v 3 reactivity was observed in celery asymptomatic patients.

### Elucidation of LTP cross-reactivity in a mouse model

**Figure 4:**
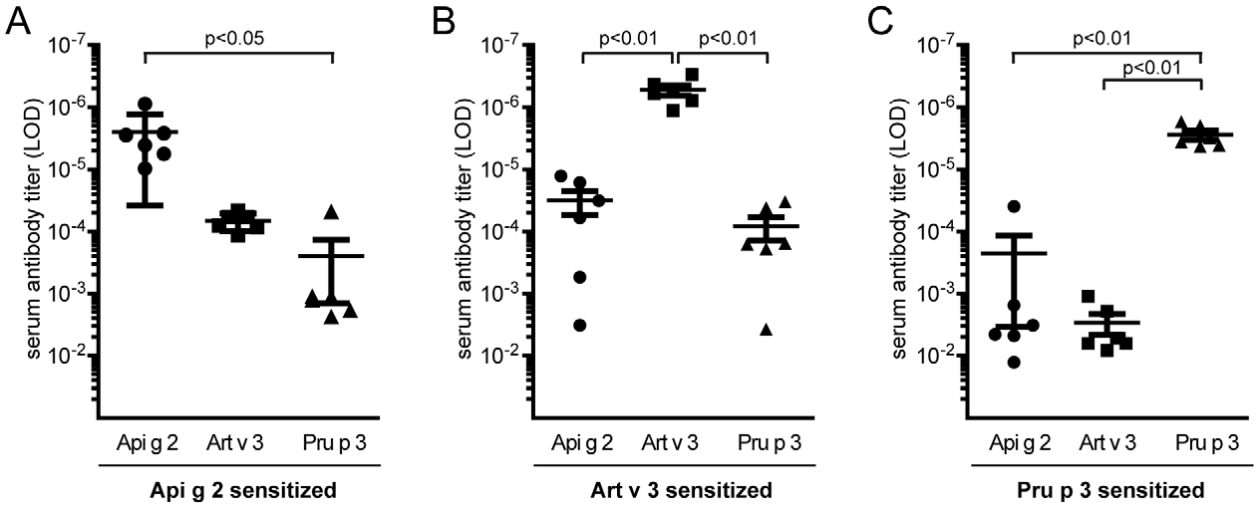
LTP cross-reactivity profiles of murine antibodies. Balb/c mice (n = 6) were immunized with purified Api g 2 (A), Art v 3 (B) and Pru p 3 (C). Allergen-specific IgG antibody titers to immobilized celery stalk, mugwort pollen and peach LTP were determined by limiting dilution in ELISA. Bars represent means and whiskers indicate standard errors of means. LOD, limit of detection.

**Figure 5:**
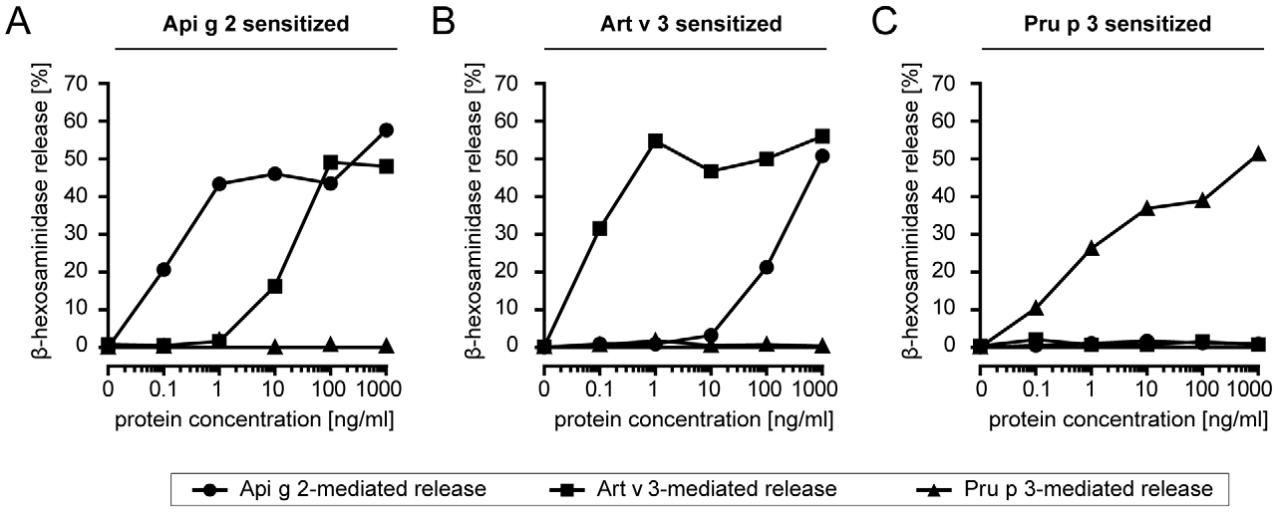
Functional IgE cross-reactivity of LTP sensitized mice. Rat basophil leukemia cells (RBL) were passively sensitized with serum pools from mice immunized with Api g 2 (A), Art v 3 (B), and Pru p 3 (C). Mediator release was triggered by serial dilutions of celery stalk, mugwort pollen and peach LTP (0.1–1000 ng/ml) and is given as percentage of total release obtained by Triton X-100.

To evaluate the development of cross-reactive antibodies under LTP mono-sensitization conditions, mice were immunized with purified single molecules. Art v 3-sensitized animals frequently developed high IgG antibody titers to both, Api g 2 and Pru p 3. Immunization with Api g 2 resulted in antibodies that were able to bind to Art v 3, while Pru p 3 sensitized mice did not develop substantial IgG titers that could recognize other LTP (Figure 4A–C). To determine IgE antibody cross-reactivity in a functional assay, degranulation of rat basophil leukemia (RBL) cells was examined after passive sensitization with serum pools of 6 mice (Figure 5A–C). Murine IgE antibodies generated upon Api g 2 and Art v 3 sensitization showed biologically relevant cross-reactivity. By contrast, Pru p 3 was not able to trigger a mediator release in celery stalk and mugwort pollen LTP sensitized mice (5A-B). Notably, IgE-mediated degranulation using sera of Pru p 3-immunized animals was clearly restricted to the sensitizing molecule (Figure 5C).

### Mimicking the generation of T cell peptides by endolysosomal degradation

**Figure 6:**
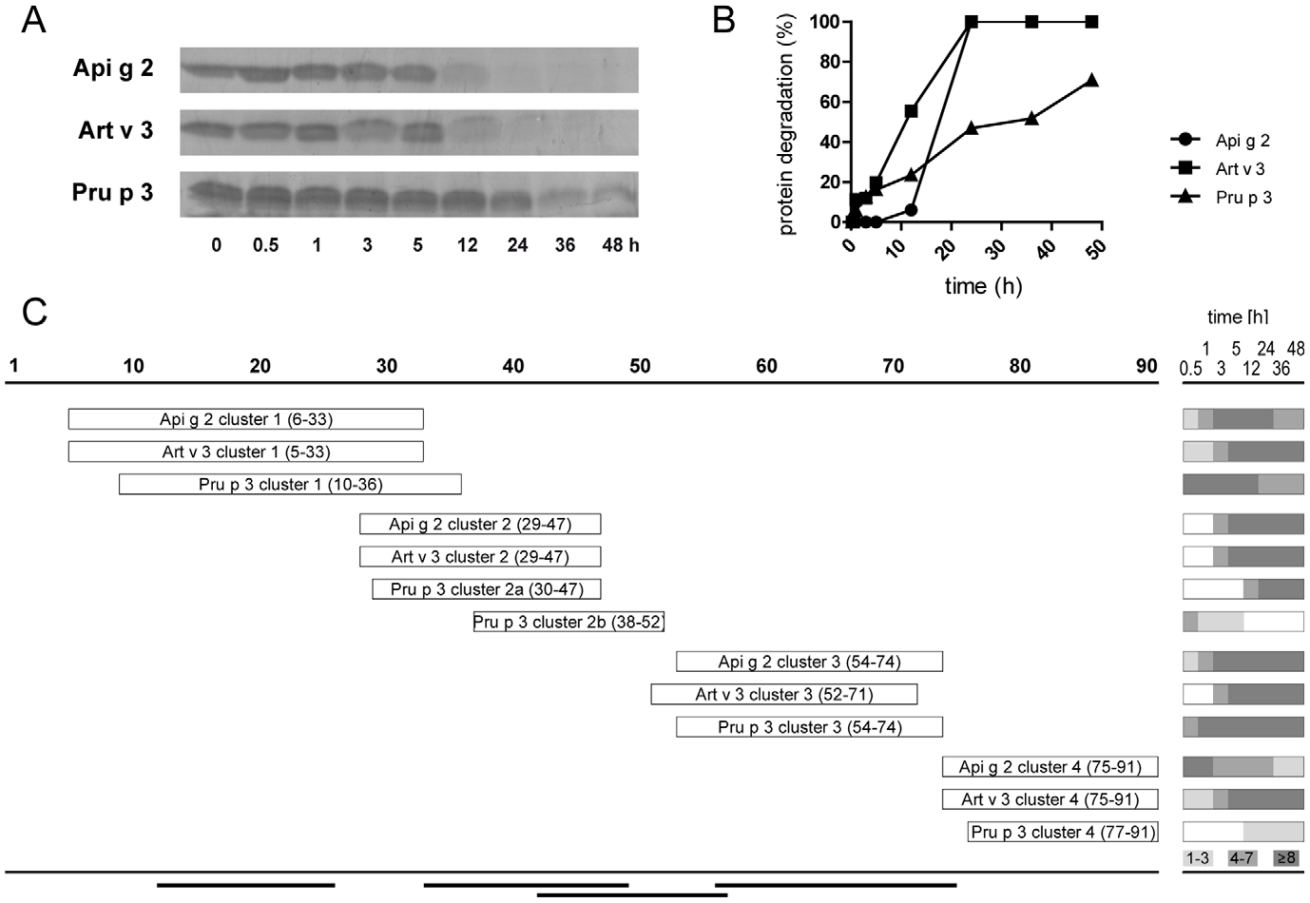
Simulated endolysosomal degradation of LTPs. Celery, mugwort pollen and peach LTP were incubated with a microsomal fraction obtained from dendritic cells of LTP allergic patients. Decline of intact proteins was monitored on SDS-PAGE (A) and evaluated densitometrically (B). Mass spectrometry based analysis of peptides demonstrated distinct clusters at similar regions of the respective LTPs and amino acid positions are given in parenthesis (C). The panel on the right side shows the number of peptides (indicated by different grey shadings) observed for each cluster during endolysosomal digestion (0.5–48 hours). Black bars on bottom represent previously identified T cell epitopes of Pru p 3 [28], [29].

To evaluate the patterns and kinetics of degradation by endolysosomal proteases simulating antigen processing, LTPs were incubated with microsomal contents isolated from dendritic cells of LTP-sensitized patients. Highest stability was observed for Pru p 3, which presented detectable intact protein in gel electrophoresis even after 48 hours of incubation. In contrast, the majority of Art v 3 and Api g 2 was proteolytically cleaved within 12 hours (Figure 6A). Protein degradation was densitometrically evaluated and calculated half-lives were 11, 18, and 32 hours for Art v 3, Api g 2, and Pru p 3, respectively (Figure 6B). Generated peptides were identified by tandem mass spectrometry and individually aligned to the amino acid sequence of celery (Figure S1), mugwort (Figure S2), and peach (Figure S3) LTP. In general, the highest overall numbers of peptides were obtained for Api g 2 and Art v 3 which correlates with their higher proteolytic susceptibility. Although LTP demonstrate only moderate primary sequence identity, all proteins presented four distinct peptide clusters spanning similar regions. However, peptides within those defined clusters differed regarding their numbers as well as kinetics of appearance (Figure 6C). Cluster 1 and 3 comprised the largest numbers of peptides which were also generated continuously throughout the incubation period. A later onset was observed for peptides within cluster 2, a subset of early generated peptides (Pru p 3 cluster 2b) was exclusively observed for peach LTP. Interestingly, Api g 2 and Art v 3 produced considerable numbers of peptides comprising cluster 4, while only a limited number could be assigned to the C-terminal region of Pru p 3. As depicted in Figure 6C, peptide regions generated by endolysosomal proteases perfectly matched previously identified T cell epitopes of Pru p 3 [28], [29], [30]. Interestingly, cluster 1 and 3 which comprised most of the peptides were also shown to harbor the immunodominant and most important T cell epitopes within amino acids 12–27 and 57–75 of Pru p 3 [28], [29].

## Discussion

In the Mediterranean area, up to 60% of patients allergic to fruits and/or vegetables display LTP-specific IgE antibodies [31]. Sensitization to Pru p 3, the most important allergen trigger in this protein family is associated with an increased risk in the prevalence as well as in the severity of food allergies [5]. With the increasing number of identified allergenic LTP (www.allergome.org), cross-reactivity and sensitization pattern can be determined in more detail. In this study we investigated the relevance of Api g 2 [8], the recently characterized nsLTP1 from celery stalks in a Mediterranean population. Interestingly, the sensitization prevalence to Api g 2 was only slightly lower than that observed for Pru p 3, and higher than that of Art v 3 and Cor a 8, the nsLTP of hazelnut (15.8%, unpublished data) in our 786 tested subjects. Although diverse sensitization patterns were observed similar to previous studies, [7], the majority of the individuals reacted to all 3 investigated LTP and we found a good correlation of specific IgE levels. Interestingly, we identified several individuals reacting exclusively to one of the three LTP under investigation. Although this finding can be suggestive of mono-sensitization, this would require a broader IgE testing of LTP molecules. To analyze cross-reactivity and relevance of Api g 2 sensitization in the context of LTP pollen and food allergy, we defined a cohort of 32 patients positive to Api g 2 with *in vitro* reactivity to Art v 3 and Pru p 3. Among this population we observed 10 patients with clinically relevant celery allergy, suggesting that Api g 2 might represent an important *Apium graveolens* allergen in the LTP-sensitized Mediterranean population. In contrast to Central and Northern European birch-endemic areas where sensitization to Api g 1 and Api g 4 is predominant [13], [18], these celery allergens were of negligible relevance in our investigated cohort and only one of the symptomatic patients demonstrated reactivity to this celery components. Generally, the sensitization frequency to Api g 1 is rather low in Italy (1.6%) [6], which can be explained by the fact that there is low exposure to birch trees. In addition, the Mediterranean population almost exclusively consumes celery stalks which contain only a marginal amount of Api g 1 compared to celery tuber (unpublished data). All those facts suggest that Api g 2 could be involved in celery stalk allergy in our patients' cohort, however the demonstration of clinical relevance regarding this molecule demands further investigation. Although sensitization to LTP was consistently reported in the context of development of severe symptoms [1], these molecules predominately elicit mild local symptoms [3]. Likewise, the majority of our patients displayed OAS after consumption of LTP-containing food, but some were also affected by more severe and systemic symptoms. Interestingly, sera of symptomatic patients frequently displayed a more pronounced self-inhibition to Api g 2 thus highlighting the importance of antibody binding affinity over quantitative levels of specific IgE. Api g 2 demonstrates similar resistance to gastric and thermal treatment as Pru p 3 [4], [8], [9], therefore it may also possess the capacity to trigger generalized allergic reactions and sensitize via the gastrointestinal tract.

Due to their conserved alpha-helical structure, LTP are involved in varying degrees of IgE cross-reactivity with different levels of clinical relevance [3], [7]. In a preceding study, we showed that thermal treatment of Api g 2 at low concentrations (4 µg/ml) did not significantly influence the ability to bind human IgE [8]. However, denaturation of LTP at concentrations of 1-2 mg/ml in neutral buffers completely abolished IgE reactivity to Api g 2 and Art v 3 in the present study. This observation might be explained by the fact that high temperatures at neutral pH can lead to disulfide bond cleavage facilitating generation of inter-molecular cysteine-linkages. This might in turn favor aggregate formation which seems to be linked to the concentration of the molecule used during denaturation. Generally, it can be anticipated that the majority of LTP epitopes are dependent on an intact 3-dimensional fold as loss of the disulfide stabilized structure by reduction and alkylation resulted in complete unfolding of Pru p 3 and subsequent loss of allergenicity [32]. Even though Pru p 3 was able to maintain minor parts of its IgE-binding activity upon treatment at 95°C in our study, this might be due to higher resistance to thermal processing which could have left some conformational epitopes still intact. In previous studies, several IgE-binding epitopes of Pru p 3 were identified by peptide and mimotope mapping [25], [26], [27]. However, the relevance of IgE-binding to linear epitopes remains unclear, given the fact that unfolding of Pru p 3 led to completely abrogated allergenicity [32]. Nevertheless, highest sequence identities of Pru p 3 to Api g 2 and Art v 3 are found in the C-terminal region which could therefore constitute a generally LTP cross-reactive epitope (Figure 2). The identified N-terminal epitope seems to be less conserved and likely represents a *Rosaceae*-specific epitope [25], with lower homology to LTP from other families. A striking sequence similarity between Api g 2 and Art v 3 was observed within residue 27–44, which might encompass a cross-reactive celery stalk/mugwort epitope thus explaining the higher inhibitory capacity of Api g 2 to mugwort when compared to peach LTP. Interestingly, the IgE-binding to Art v 3 was not inhibited by Pru p 3 in 3/20 patients' sera, suggesting that these individuals were primarily sensitized to mugwort pollen Art v 3 and IgE reactivity to Pru p 3 might be due to cross-reactivity. These patients were also reactive to Art v 1, a genuine marker allergen for *Artemisia* sensitization [33], which further corroborates this idea. The distinction between LTP co-sensitization and cross-reactivity is generally difficult, explaining the contradictory results and divergent interpretation of clinical manifestations in previous studies [11], [12]. As Art v 3 and Pru p 3 seem to harbor common but also distinct allergen-specific IgE-epitopes, both pollen and/or food LTP were suggested as primary sensitizer [3]. Since the sensitizer question cannot be unequivocally answered in human studies, we used a mouse model to investigate antibody cross-reactivity under mono-sensitization conditions. By immunizing purified recombinant molecules, biased antibody responses due to trace amounts of allergen impurities in a natural LTP preparation (e. g. profilins, PR-10 proteins) could be entirely ruled out [8]. Based on our *in vivo* data, immunization with Api g 2 or Art v 3 seems to induce antibodies that were able to recognize the homologous LTP of mugwort and celery stalks, and furthermore trigger biologically relevant mediator release via IgE cross-linking. Notably, antibodies raised against Pru p 3 were not able to trigger mediator releases when tested with Api g 2 and Art v 3. Results obtained from this murine model cannot be unequivocally translated to the human situation, e. g. due to diverse HLA repertoires. However, it remains still unclear if only one particular LTP is involved in primary sensitization, since we identified a considerable number of individuals reacting to Art v 3 and/or Api g 2 in the absence of a Pru p 3 sensitization. Therefore, one might speculate that LTP allergy could coexist as at least two independent branches, (i) sensitization via food Pru p 3 and (ii) sensitization via pollen, e.g. Art v 3. Such a multi-sensitization may result in broadening of the spectrum of IgE reactivity to different LTP, while sensitization could also take place through an LTP that was not yet identified. The fact that some studies showed that pollen LTP can act as primary sensitizer [11], [34], [35], [36] is supporting the concept and may help to understand contradictory study outcomes of food and pollen LTP [7]. This idea is further supported by our mouse data which showed Pru p 3 to be independent of Art v 3 and Api g 2 antibody cross-reactivity.

The previously reported association of sensitization to *Apium graveolens* and *Artemisia vulgaris* pollen [10] could be partially attributed to the IgE cross-reactivity between Api g 2 and Art v 3. However, commercially available products for allergy diagnosis often do not indicate the specific tissue of *Apium graveolens* ingredients used for extract preparation. Notably, there is a tremendous difference regarding tissue expression of Api g 2. The protein was only present in the aerial tissue but could not be detected in tuber, a fact that could be easily overcome using molecule-based allergy diagnosis [14]. However, the involvement of Api g 2 in the celery-mugwort syndrome described for Central European patients with allergic reactions to celeriac is not yet clear and needs further investigations in a clinical study.

It has previously been shown that protein stability affects both, allergenicity and immunogenicity of allergens. Susceptibility to endolysosomal proteolysis has been demonstrated to correlate with immunogenicity, as rather stable molecules warrant continuous supply of T cell stimulatory peptides [37], [38]. All investigated LTP displayed relatively long half-lives of >10 hours, and thus were more resistant to degradation as *e. g.* Bet v 1 from birch pollen [37]. Observed differences in the susceptibility to endolysosomal degradation might be explained by the primary sequence of investigated LTP. In particular the varying number of surface exposed lysine residues, i. e. Pru p 3 (4 lysines), Api g 2 (8 lysines), Art v 3 (13 lysines) which are susceptible to Cathepsin S cleavage, an important protease for antigen processing [39] may influencing the cleavage efficiency. Interestingly, stability to endolysosomal degradation correlated with stability to gastric digestion and thermal treatment of investigated LTP [8], [40]. Generally, Pru p 3 can be considered the most stable, while the pollen LTP Art v 3 seems to be less stable than plant food LTP. Mass spectrometry-based analysis of endolysosomal degradation revealed that all three LTP share similar proteolytic sites, which is reflected by the occurrence of four common nested clusters of peptides sharing a central core with variable flanking residues spanning identical protein regions. These data indicate a strong influence of the three-dimensional protein structure on the generation of T cell epitopes. In general, Api g 2 and Art v 3 displayed a more congruent profile compared to Pru p 3, particularly in terms of peptide numbers, kinetics, and the absence of sub-cluster 2b which was only observed for Pru p 3. Interestingly, the i*n vitro* generated peptides of Pru p 3 (cluster 1–3) perfectly matched to recently identified T cell epitopes [28], [29]. The importance of cluster 3 was also unraveled in two recent studies using natural Pru p 3 and microsomes from human or murine dendritic cells [32], [41]. Notably, this cluster harbors the immunodominant T cell epitope Pru p 3_61–75_ and it is characterized by an early onset and long-lasting peptide generation. Fragments within clusters 2b and 3 were recently also found upon simulated duodenal digestion of Pru p 3 [42], suggesting the presence of T cell stimulatory peptides directly on site of the intestine. Interestingly, the C-terminal cluster of Pru p 3 contained only few peptides and seems to be irrelevant for its T cell stimulation [29]. In contrast, numerous peptides of Api g 2 and Art v 3 were found within cluster 4, a fact that was recently also observed for Cor a 8 [41], suggesting a possible relevance in T-cell reactivity for celery, mugwort pollen and hazelnut.

In the present study we could show that Api g 2 represents a relevant, cross-reactive allergen of *Apium graveolens* in patients suffering from LTP-mediated allergy in the Mediterranean area. Api g 2 contains common B cell epitopes of both, pollen and food-derived LTPs. However, data obtained from mouse immunization experiments revealed that Api g 2 shares more epitopes with mugwort Art v 3 and seems to be rather distinct from peach Pru p 3. Generally, the number and origin of LTP that patients react to is highly inhomogeneous, and the distinction between co-sensitization and cross-reactivity is rather difficult. Although, it is commonly accepted that food LTP, i. e. Pru p 3 acts as primary sensitizer in LTP allergy, our data indicate that in accordance with classical pollen-associated food allergies, sensitization might in addition be mediated by pollen LTP.

## Materials and Methods

### Screening for IgE reactivity to Art v 3, Api g 2, and Pru p 3 in an Italian sensitized population

786 non-randomly distributed allergic subjects were selected on the basis of at least one positive IgE result on the ISAC 103 microarray (PMD, Vienna, Austria) [6]. Subjects were tested on a customized microarray including recombinant Art v 3 and Api g 2 [8], [40] and natural Pru p 3 [43]. Purified proteins were immobilized on the microarray and tested following the established ISAC testing procedures [6].

### Clinical evaluation of a selected cohort

For detailed analysis, we selected a patient's cohort (n = 32) presenting inhalation- and/or ingestion related allergy symptoms to LTP containing pollen or food sources with IgE sensitization to Api g 2, Art v 3, and Pru p 3. Clinical features of patients were recorded using InterAll *e*-record (Allergy Data Laboratories s.c., Latina, Italy). The study was approved by the Institutional Review Board of Istituto Dermopatico dell'Immacolata – IDI-IRCCS, Rome, Italy (n. 106-CE-2005), and signed informed consents were obtained.

### Recombinant production of LTP from celery stalks, mugwort pollen, and peach fruit

The sequence corresponding to the mature celery LTP (GenBank accession no. FJ643539) was cloned into the pHis Parallel2 expression vector. Recombinant Api g 2.0101 was expressed as soluble non-fusion protein in *E. coli* Rosetta-gami B(DE3) pLysS (Novagen, Gibbstown, NJ) according to established protocols [8], [40]. Briefly, protein purification was performed with ion exchange chromatography using a SP FF column (GE Healthcare, Chalfont St. Giles, UK) and subsequently submitted to gel filtration on a Superdex 10/300 GL column (GE Healthcare). Details on the molecular characterization and demonstration of equivalent physicochemical and immunological properties of the natural and recombinant molecule are reported elsewhere [8]. Recombinant Art v 3.0201 [40] and Pru p 3 [44] were produced and purified as previously described. Purified proteins were analyzed by denaturing SDS-PAGE and visualized by Coomassie Brilliant Blue R-250 staining. Protein concentrations were determined using the Pico Tag method (Waters, Milford, MA) and circular dichroism measurements were performed at 20°C to demonstrate the structural integrity of all proteins following published protocols [8], [40].

### ELISA and inhibition assays

Sera of 32 patients were tested for IgE reactivity to rApi g 2, rArt v 3, and rPru p 3 in ELISA experiments. Maxisorp plates (Nalge Nunc, Rocherster, NY) were coated with 200 ng allergen in PBS o/n at 4°C. Unspecific binding was blocked with TBS pH 7.4, 0.05% (v/v) Tween-20 and 1% (w/v) BSA and incubated with 1∶4 diluted patients' sera o/n at 4°C. Bound IgE was detected with alkaline phosphatase-conjugated monoclonal anti human IgE antibodies (BD Biosciences, Franklin Laker, NJ). Specific serum IgE levels were calculated based on a quantitative sandwich ELISA using goat anti-human IgE antibody (KPL, Gaithersburg, MD) and purified human IgE (Serotec, Raleigh, NC) as internal standard. Measurements were performed in duplicates and values exceeding 3xSTD of background were considered positive. In addition, purified recombinant allergens at concentrations of 1–2 mg/ml in 50 mM phosphate buffer were heat denatured (15 min at 95°C) prior to coating on solid phase and IgE reactivity was assessed as described above.

Inhibition studies were performed as “single point highest inhibition achievable assay” in a microarray format [40]. Undiluted sera of 20 individual patients were pre-incubated overnight with purified allergens at a concentration of 0.4–0.5 mg/ml and the percentage of inhibition was determined. In addition we assessed the dose-dependent IgE inhibition to solid phase coated rApi g 2 in ELISA using sera of ten patients with different clinical manifestations to celery stalks. Cross-inhibition was performed with increasing concentrations of purified Api g 2, Art v 3, and Pru p 3 (10^−4^–10^1^ µg/ml).

### Animal experiments

Female BALB/c mice (Charles River Laboratories, Wilmington, MAS) were immunized subcutaneously with 10 µg allergen in 50 µl DPBS adsorbed to 50 µl Alugel-S (Serva, Heidelberg, Germany) given as two 50 µl subcutaneous injections administered bilaterally in the lumbar region and boosted on days 14, 28, and 42. LPS concentrations of the proteins were <3 EU/mg as determined by the limulus amoebocyte lysate assay (Pyrochrome, East Falmouth, MA). Antigen-specific total IgG levels were determined using a peroxidase-conjugated rabbit anti mouse IgG antibody (Bio-Rad, CA) followed by chromogenic substrate development and antigen titers were calculated as limit of detection (LOD) values. Functional IgE levels were measured in a mediator release assay using rat basophil leukaemia (RBL)-2H3 cells [45]. Briefly, RBL cells were passively sensitized with a 1∶40 diluted murine serum pool (n = 6) raised against rApi g 2, rArt v 3, and rPru p 3, respectively. Cross-linking and degranulation was triggered by serial protein dilutions and β-hexosaminidase release was measured by enzymatic cleavage of the fluorogenic substrate 4-methyl umbelliferyl-N-acetyl-β-glucosaminidase (Sigma). Results are reported as percentage of total β-hexosaminidase release of Triton-X100- treated cells. Animal experiments were conducted according to National guidelines approved by the Austrian Ministry of Science and Research, Ref. II/10b Genetic engineering and Animal experiments (approval Nr. BMWF-66.012/0011-II/10b/2010).

### Endolysosomal degradation assays

Microsomes were isolated from monocyte-derived dendritic cells obtained from 16 LTP-allergic donors, and used for protein degradation according to a recently established protocol from our laboratory [38]. Briefly, cells were harvested by low speed centrifugation, re-suspended in 10 mM Tris acetate pH 7.0, 250 mM sucrose and homogenized with a Dounce glass tissue homogenizer. Cellular debris and nuclei were removed by centrifugation at 6000 x *g* and microsomes isolated by ultracentrifugation at 100,000 x *g*. Protein aliquots of 5 µg were digested with 7 µg microsomal enzymes in 100 mM citrate buffer, pH 4.8 and 2 mM dithiothreitol at 37°C. Reactions were stopped by heat-denaturation at various time points and analyzed by SDS-PAGE and densitometric quantification of protein bands. Peptides obtained from microsomal digestion were separated by capillary HPLC on-line coupled to a Micromass QTof Global Ultima instrument (Waters, Mildford, MA) as recently described [37]. Individual peptides were grouped into clusters representing regions that cover the majority of generated peptides.

### Modelling

Homology modeling of Api g 2, Art v 3, and Pru p 3 was performed using Modeller 9v2 (http://salilab.org/modeller) and evaluated with ProSa2003 based on the structure of Pru p 3 (pdb: 2B5S).

### Statistical analysis

Statistical evaluation was performed using the Wilcoxon signed rank sum test and Spearman rank sum test. Groups that passed normality and equal variance tests were analyzed with paired samples *t*-test. A value of P<0.01 was considered statistically significant.

## Supporting Information

Figure S1 **Proteolytic fragments obtained from endolysosomal degradation of Api g 2.** Peptides sequenced by mass spectrometry after 0.5, 1, 3, 5, 12, 24, 36, and 48 hours of *in vitro* digestion with microsomal fractions from monocyte-derived dendritic cells of LTP-allergic patients are depicted within the mature sequence of Api g 2.
(TIF)

Figure S2 **Proteolytic fragments obtained from endolysosomal degradation of Art v 3.** Peptides sequenced by mass spectrometry after 0.5, 1, 3, 5, 12, 24, 36, and 48 hours of *in vitro* digestion with microsomal fractions from monocyte-derived dendritic cells of LTP-allergic patients are depicted within the mature sequence of Art v 3.
(TIF)

Figure S3 **Proteolytic fragments obtained from endolysosomal degradation of Pru p 3.** Peptides sequenced by mass spectrometry after 0.5, 1, 3, 5, 12, 24, 36, and 48 hours of *in vitro* digestion with microsomal fractions from monocyte-derived dendritic cells of LTP-allergic patients are depicted within the mature sequence of Pru p 3.
(TIF)
