# Supplementary figures and images for: Sensitization Prevalence, Antibody Cross-Reactivity and Immunogenic Peptide Profile of Api g 2, the Non-Specific Lipid Transfer Protein 1 of Celery
Source: PLoS One. 2011 Aug 29;6(8):e24150. doi: 10.1371/journal.pone.0024150 (PMC3163685; doi:10.1371/journal.pone.0024150)

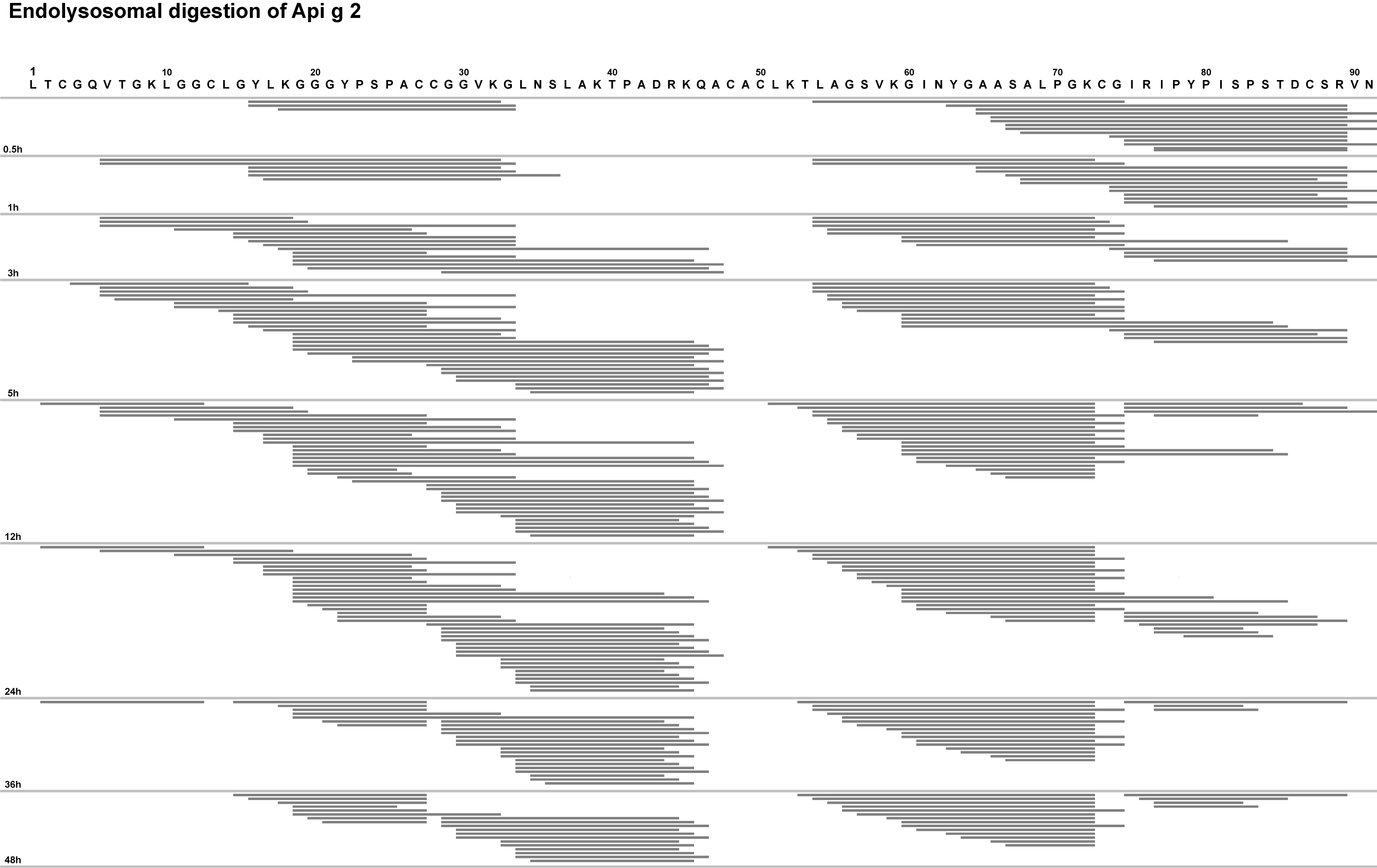

Supplement: Figure S1 — Proteolytic fragments obtained from endolysosomal degradation of Api g 2. Peptides sequenced by mass spectrometry after 0.5, 1, 3, 5, 12, 24, 36, and 48 hours of in vitro digestion with microsomal fractions from monocyte-derived dendritic cells of LTP-allergic patients are depicted within the mature sequence of Api g 2. (TIF) [file pone.0024150.s001.tif]

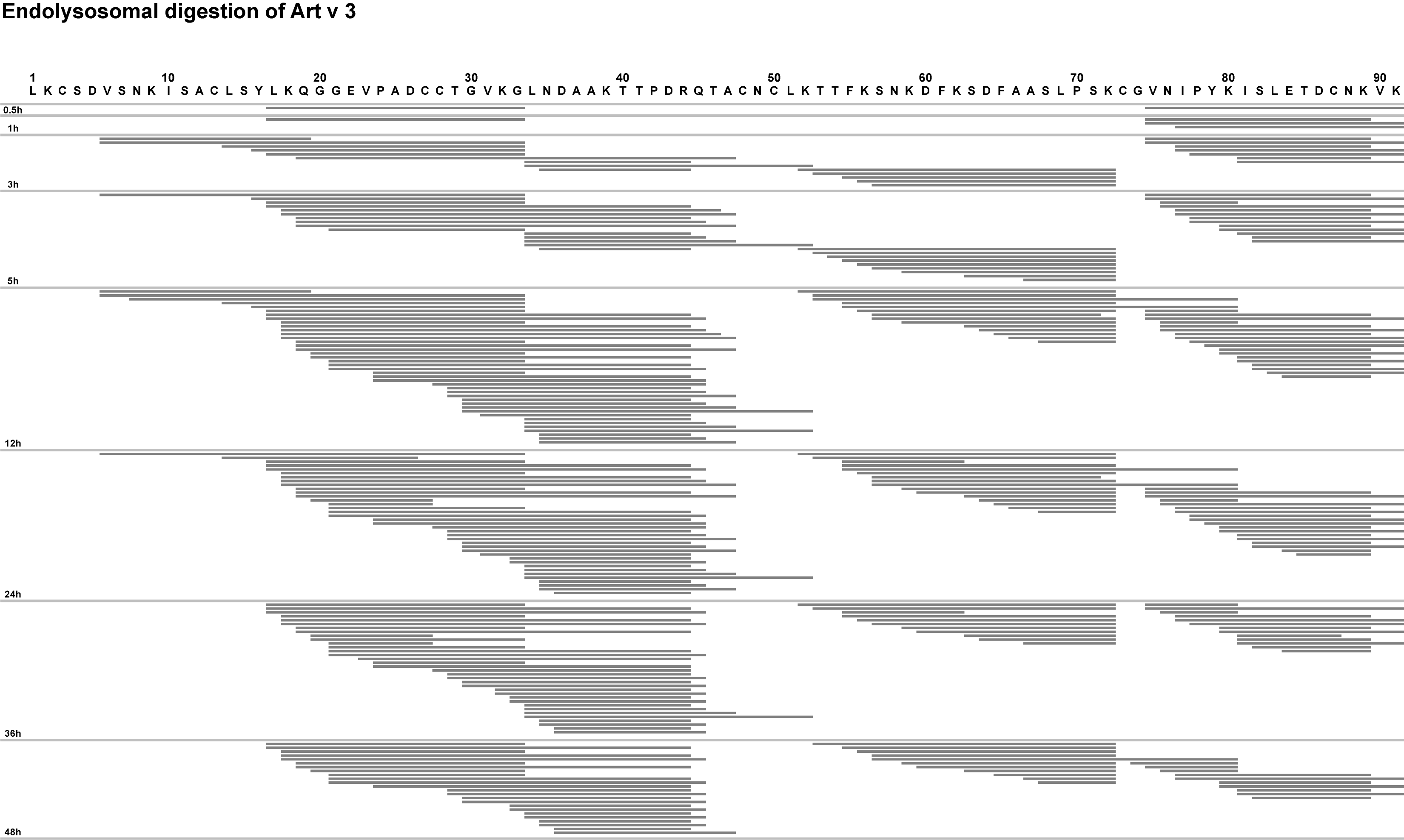

Supplement: Figure S2 — Proteolytic fragments obtained from endolysosomal degradation of Art v 3. Peptides sequenced by mass spectrometry after 0.5, 1, 3, 5, 12, 24, 36, and 48 hours of in vitro digestion with microsomal fractions from monocyte-derived dendritic cells of LTP-allergic patients are depicted within the mature sequence of Art v 3. (TIF) [file pone.0024150.s002.tif]

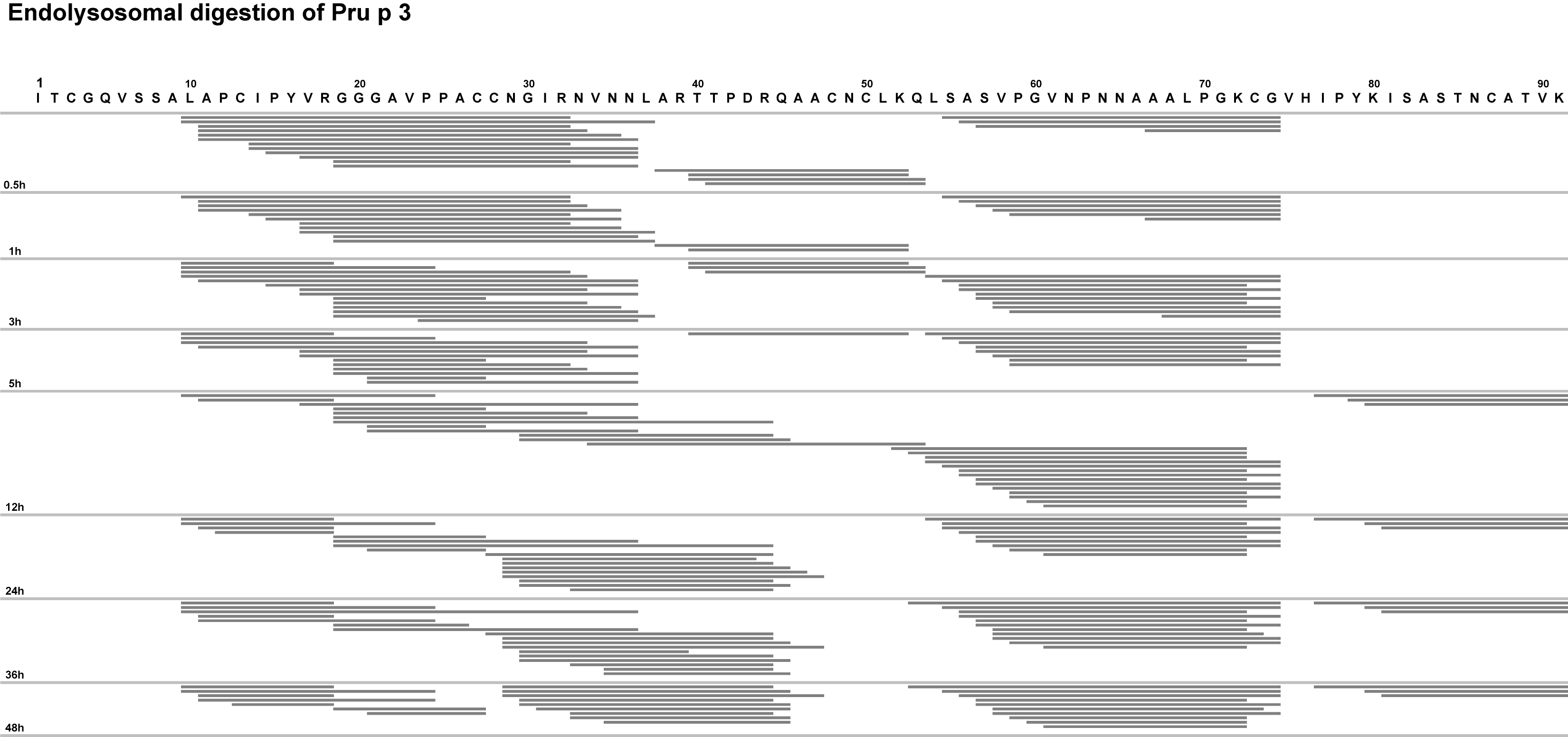

Supplement: Figure S3 — Proteolytic fragments obtained from endolysosomal degradation of Pru p 3. Peptides sequenced by mass spectrometry after 0.5, 1, 3, 5, 12, 24, 36, and 48 hours of in vitro digestion with microsomal fractions from monocyte-derived dendritic cells of LTP-allergic patients are depicted within the mature sequence of Pru p 3. (TIF) [file pone.0024150.s003.tif]
